# FROM BIG DATA TO COMMUNITY SETTINGS: HOW CAN EXERCISE IMPROVE HEALTH OUTCOMES IN OLDER CANCER SURVIVORS?

**DOI:** 10.1093/geroni/igz038.1421

**Published:** 2019-11-08

**Authors:** Shirley M Bluethmann, Corinne Leach

**Affiliations:** 1 The Pennsylvania State University College of Medicine, Hershey, Pennsylvania, United States; 2 American Cancer Society, Atlanta, Georgia, United States

## Abstract

Current guidelines recommend that survivors achieve regular physical activity and reduced inactivity to attenuate cancer and mortality risk and to promote quality of life. Optimal ways of obtaining physical activity or measuring how much exercise has been achieved continues to be a challenge, especially among older adults and cancer survivors. Our symposium will provide insights using big data to understand population level patterns of activity and potential benefits for survivors, examining implications for survival and key health outcomes, including multimorbidity and functional limitations. We will also discuss social and environmental determinants that may be important for older survivors in designing community interventions, especially in rural communities. These include considerations related to the built environment and social support to promote leisure-time physical activity in older survivors.

